# Cytokine expression and ultrastructural alterations in fresh-frozen, freeze-dried and γ-irradiated human amniotic membranes

**DOI:** 10.1007/s10561-016-9553-x

**Published:** 2016-04-12

**Authors:** Adolfo Paolin, Diletta Trojan, Antonio Leonardi, Stefano Mellone, Antonio Volpe, Augusto Orlandi, Elisa Cogliati

**Affiliations:** 1 Treviso Tissue Bank Foundation, Piazzale Ospedale 1, Via Scarpa 9, 31100 Treviso, Italy; 2 Department of Molecular Medicine and Biomedical Technologies, Medical School, Federico II University of Naples, Via Pansini 5, 80131 Napoli, Italy; 3 Anatomic Pathology, Department of Biomedicine and Prevention, Tor Vergata University of Rome, Rome, Italy

**Keywords:** Amniotic membrane, Cytokines, Freeze-drying, γ-Irradiation, Transmission electron microscopy

## Abstract

The aim of this work was to compare the effects on human amniotic membrane of freeze-drying and γ-irradiation at doses of 10, 20 and 30 kGy, with freezing. For this purpose, nine cytokines (interleukin 10, platelet-derived growth factor-AA, platelet-derived growth factor-BB, basic fibroblast growth factor, epidermal growth factor, transforming growth factor beta 1, and tissue inhibitors of metalloproteinase-1, -2, and -4) were titrated in 5 different preparations for each of 3 amniotic membranes included in the study. In addition, the extracellular matrix structure of each sample was assessed by transmission electron microscopy. While freeze-drying did not seem to affect the biological structure or cytokine content of the different amniotic membrane samples, γ-irradiation led to a significant decrease in the tissue inhibitors of metalloproteinase-4, basic fibroblast growth factor and epidermal growth factor, and induced structural damage to the epithelium, basement membrane and lamina densa. The higher the irradiation dose the more severe the damage to the amniotic membrane structure. In conclusion, the Authors recommend processing amniotic membrane under sterile conditions to guarantee safety at every step rather than final sterilization with γ-irradiation, thereby avoiding alteration to the biological characteristics of the amniotic membrane.

## Introduction

Human amniotic membrane (HAM) has been used in a variety of surgical procedures. First employed in skin transplantation by (Davis 1910), HAM was subsequently found to be useful as a biological wound dressing for burns (Ramakrishnan and Jayaraman 1997; Branski et al. 2008), acute (Tekin et al. 2008) and chronic wounds (Gajiwala and Lobo 2003; Insausti et al. 2010), and in the reconstruction of the dura mater (Tomita et al. 2012; De Weerd et al. 2013), oral cavity (Lawson 1985), vaginal vault (Ashworth et al. 1986), tendons (Ozbölük et al. 2010) and nerves (O’Neill et al. 2009). HAM has also long been used in ophthalmic surgery, the earliest reported application being in 1940 when De Rötth used fetal membranes to correct symblepharon (De Rötth 1940). Today HAM is widely used for ocular surface reconstruction and treating several important ocular diseases (Paolin et al. 2016). All these applications are possible because HAM has anti-inflammatory, antifibrotic properties (Solomon et al. 2001; Tseng et al. 1999).

Hao et al. have shown that human amniotic epithelial and mesenchymal cells both express interleukin-1 receptor antagonist, all the four tissue inhibitors of metalloproteinase (TIMPs), collagen XVIII, and interleukin-10 (Hao 2000). Moreover, reverse transcriptase–polymerase chain reaction (RT-PCR) has shown that HAM expresses several additional cytokines, such as transforming growth factor (TGF-α, -β1, -β2), epidermal growth factor (EGF), keratinocyte growth factor (KGF), basic fibroblast growth factor (bFGF), keratinocyte growth factor receptor (KGFR), hepatocyte growth factor (HGF) and hepatocyte growth factor receptor (HGFR) (Koizumi et al. 2000; Li et al. 2005; Gicquel et al. 2009).

Since these factors may contribute to the clinical outcomes of HAM implants, several studies have endeavored to evaluate the effects of HAM storage conditions on their content and cell viability (Hennerbichler et al. 2007, Wolbank et al. 2009).

To date, the methods adopted for HAM storage are freezing at −80 °C (Mermet et al. 2007) or at −196 °C in liquid nitrogen vapor (Alió et al. 2005), and freeze-drying (Rahman et al. 2009; Riau et al. 2010).

In 2001, Adds et al. reported no differences in terms of clinical results between fresh and frozen HAM, both preparations resulting in improved visual acuity. Furthermore, fresh tissue performed no better than frozen tissue in promoting re-epithelialization (Adds et al. 2001). It has, however, been demonstrated that different processing, storage and sterilization methods do affect HAM properties. (von Versen-Höynck et al. 2004).

Rodriguez-Ares et al. studied the effects of freeze-drying and cryopreservation on HAM histological characteristics and protein levels. The authors found that although lyophilization does not affect the histological structure of HAM, it seems to reduce growth factor concentration more than cryopreservation (Rodriguez-Ares et al. 2009). Ricci et al. demonstrated that cryopreservation maintains the anti-fibrotic properties of HAM when used as a patch to reduce the severity of liver fibrosis (Ricci et al. 2013). Freeze-dried HAM does, however, have the advantage of allowing storage and shipment at room temperature, making handling much easier.

The aim of this study was to evaluate the effect of γ-irradiation on cytokine levels and the ultrastructure of the extracellular matrix of different HAM preparations.

Accordingly, we a) carried out a quantitative measurement of the following cytokines: interleukin 10 (IL-10), platelet-derived growth factor-AA (PDGF-AA), platelet-derived growth factor-BB (PDGF-BB), basic fibroblast growth factor (bFGF), epidermal growth factor (EGF), transforming growth factor beta 1 (TGF-β1), tissue inhibitors of metalloproteinase-1 (TIMP-1), -2 (TIMP-2), and -4 (TIMP-4), and b) analyzed each HAM preparation with transmission electron microscopy.

## Materials and methods

### HAM collection and processing

Three placentas were sourced from elective cesarean sections after obtaining written informed consent in hospitals belonging to our tissue bank procurement network. Donors were selected on the basis of strict criteria that also include guidelines for harvesting, processing and distributing tissues for transplantation as approved by the National Transplant Centre. Selection criteria included the absence of any kind of malignancy, infant malformation or pathology, a gestation period of at least 35 weeks, negative family medical history for genetic diseases, and lifestyles of both parents not at risk for infectious diseases. On arrival at the bank the tissues were anonymized with a unique code number used for all processing phases.

Working in sterile conditions in a laminar flow cabinet within 24 h of tissue retrieval, HAM was carefully detached from the chorion and rinsed with saline solution to remove blood clots.

After processing, HAM underwent microbiological testing to ascertain its sterility and was frozen at -80 °C without cryprotectant. Prior to the study the HAM specimens were thawed, rinsed in saline solution and sterile water. Each HAM specimen was cut into 5 samples referred to as follows: a) “fresh-frozen” (one sample): left unprocessed, b) “freeze-dried” (one sample): freeze-dried, and c) “γ-irradiated” (3 samples): freeze-dried and sterilized with γ-irradiation at doses of 10, 20 and 30 kGy respectively.

The study design was not submitted to our ethical committee as consent had already been given for both clinical and research purposes.

### Cytokine quantitative assessment

A specific enzyme-linked immunosorbent assay (ELISA) kit (R&D Human Immunoassay) was used for each cytokine, as shown in Table 1.

**Table 1:** Assay kit used for each cytokine analysis

Cytokine	ELISA kit
IL-10	Quantikine ELISA–human IL-10 immunoassay, R&D catalog number D1000B
PDGF-BB	Quantikine ELISA human PDGF-BB immunoassay, R&D catalog number DBB00
PDGF-AA	Quantikine ELISA human/mouse PDGF-AA immunoassay, R&D catalog number DAA00B
bFGF	Quantikine ELISA human FGF basic immunoassay, R&D catalog number DFB50
TGF-β1	Quantikine ELISA human TGF-β1 immunoassay, R&D catalog number DB100B
TIMP-1	Quantikine ELISA human TIMP-1 immunoassay, R&D catalog number DTM100
TIMP-2	Quantikine ELISA human TIMP-2 immunoassay, R&D catalog number DTM200
TIMP-4	Quantikine ELISA human TIMP-4 immunoassay, R&D catalog number DTM400
EGF	Quantikine ELISA human EGF immunoassay, R&D catalog number DEG00

The assay employs the quantitative sandwich enzyme immunoassay technique whereby an antibody specific for each cytokine is pre-coated onto a microplate. The analysis was performed twice for each cytokine, in triplicate. Samples were re-suspended in Triton X-100 lysis buffer (20 mM Hepes, pH 7.4, 150 mM NaCl, 10 % glycerol, 1 % Triton X-100, and Complete Protease Inhibitor mixture) and placed on ice for 30 min, after which, the extracts were centrifuged for 30 min at 14,000 × g at 4 °C to remove debris before performing the ELISA assays. The ELISA assays were performed in compliance with the manufacturer’s instructions. The total protein content of each sample was determined by the Bradford protein assay and used to normalize the total cytokine concentration.

### Microscopic and ultrastructural analysis

Small formalin-fixed HAM samples were embedded in paraffin and stained with Hematoxylin-Eosin (Orlandi et al. 2005). For transmission electron microscopy, small reconstituted samples were fixed overnight in Karnovsky fixative containing 2 % glutaraldehyde, 2 % paraformaldehyde in 0.1 M sodium cacodylate buffer (pH 7.4), post-fixed in 1 % OsO4 for 2 h and dehydrated through an alcohol series and propylene oxide before embedding in EPON 812, as reported (Spagnoli et al. 1995). Ultrathin sections were cut with an 8800 ultramicrotome III (LKB, Bromma, Sweden), counterstained with uranyl acetate and lead citrate, and studied under a Hitachi electron microscope.

### Statistical analysis

Student’s *t* test was used to compare the different cytokine levels present in the different HAM preparations. Statistical significance was set at *p* < 0.05.

## Results

### Quantitative cytokine measurements

**Fig. 1:**
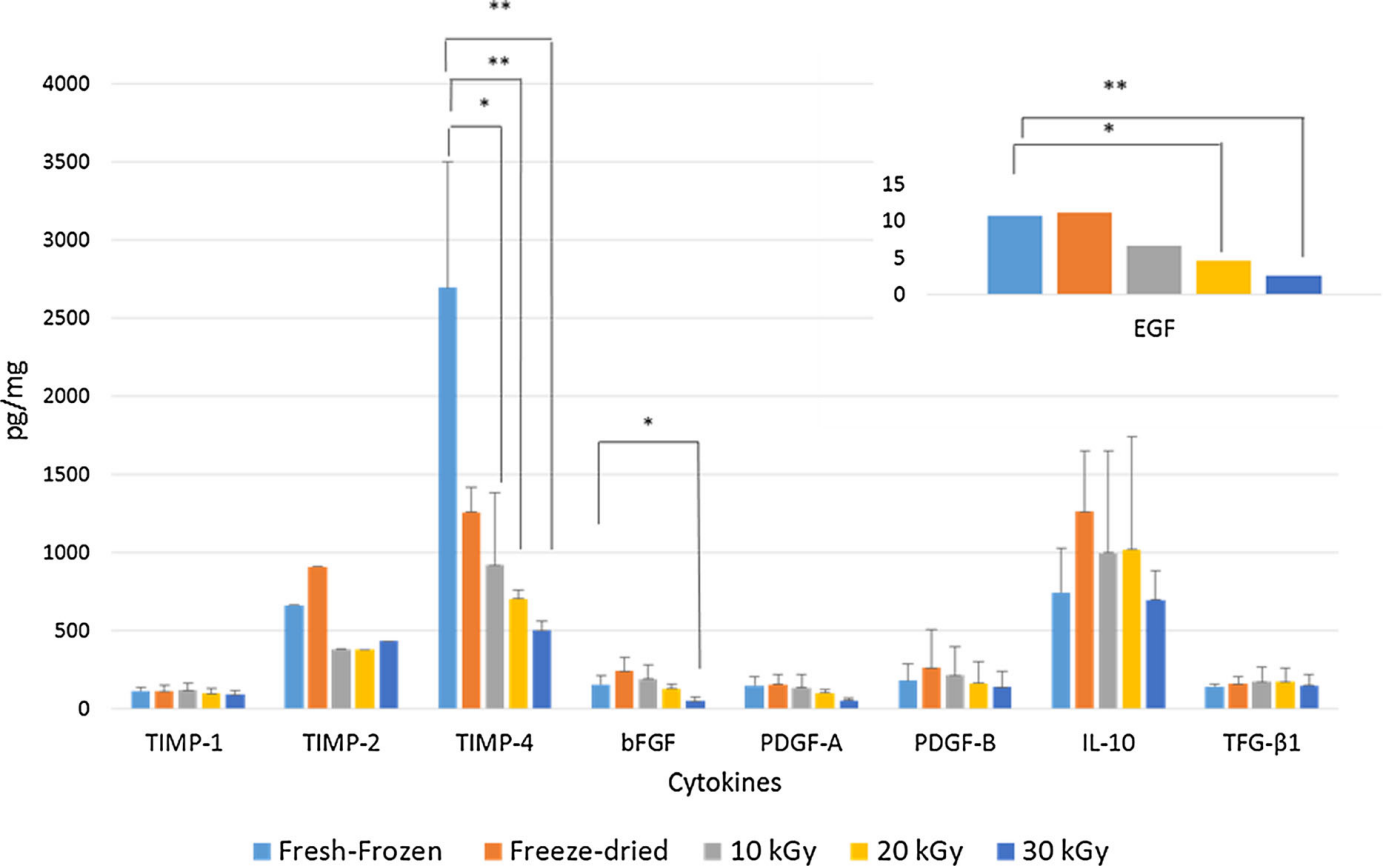
Cytokine concentrations. Cytokine concentrations in different preparations of HAM samples: fresh-frozen, freeze-dried, and sterilized with 10–20–30 kGy γ-irradiation. * indicates *p* < 0.05, ** indicates *p* < 0.01

The numerical content of cytokines in pg/mg for each HAM preparation and their percentage variations versus fresh-frozen samples are shown in Table 2. Figure 1 presents these data in histogram form.

**Table 2:** Cytokine concentrations in the different preparations

Preparation	Cytokine concentration (pg/mg)								
	TIMP-1	TIMP-2	TIMP-4	bFGF	PDGF-AA	PDGF-BB	EGF	IL-10	TFG-β1
Fresh-frozen	111.7	661.0	2692.9	153.3	146.1	176.3	10.8	742.1	138.0
Freeze-dried	109.8 (−2 %)	906.5 (+37 %)	1257.6 (−53 %)	239.4 (+56 %)	153.8 (+5 %)	257.5 (+46 %)	11.2 (+4 %)	1259.5 (+70 %)	158.7 (+15 %)
10 kGy γ-irradiated	116.2 (+4 %)	378.7 (−43 %)	917.9 (−66 %)	188.3 (+23 %)	131.6 (−10 %)	210.4 (+19 %)	6.6 (−38 %)	995.4 (+34 %)	169.3 (+23 %)
20 kGy γ-irradiated	93.1 (−17 %)	376.6 (−43 %)	701.2 (−74 %)	124.8 (−19 %)	98.9 (−32 %)	160.3 (−9 %)	4.6 (−57 %)	1017.1 (+37 %)	172.3 (+25 %)
30 kGy γ-irradiated	87.3 (−22 %)	429.7 (−35 %)	501.3 (−81 %)	47.5 (−69 %)	51.4 (−65 %)	136.3 (−23 %)	2.6 (−76 %)	693.8 (−7 %)	145.9 (+6 %)

Percentage changes in cytokine content compared to fresh-frozen samples given in brackets

Compared to fresh-frozen samples, TIMP-1 and TIMP-2 levels were not significantly affected either by freeze-drying or irradiation, even though the 30 kGy γ-irradiated HAMs showed a 22 % fall in TIMP-1 and a 35 % decrease for TIMP-2 levels. Moreover, the fall in TIMP-1 content was observed in only one of the three samples and was not statistically significant. Compared to fresh-frozen HAM, TIMP-4 was significantly lower (−66 %) in 10 kGy-irradiated HAM samples (*p* < 0.05*), and in 20 and 30 kGy irradiated HAMs (*p* < 0.01**; −74 and −81 % respectively).

The highest γ-irradiation dose caused a 69 %, statistically significant, decrease in bFGF (*p* < 0.05*) versus fresh-frozen samples, whereas low-dose irradiation and freeze-drying did not significantly affect bFGF content in any HAM preparation.

EGF levels fell significantly by 57 and 76 % respectively following 20 kGy (*p* < 0.05*) and 30 kGy (*p* < 0.01**) irradiation, in contrast to the lowest-dose irradiation and freeze-drying, which did not significantly affect EGF levels compared to fresh-frozen samples.

Compared to the fresh-frozen samples, PDGF-AA and PDGF-BB levels were not significantly affected by either freeze-drying or irradiation, even if 30 kGy γ-irradiated HAM samples were found to have 65 % less PDGF-AA and 23 % less PDGF-BB compared to the fresh-frozen samples.

Lastly, IL-10 and TGFβ-1 concentrations were not significantly affected either by irradiation or freeze-drying in any samples.

### Ultrastructural analysis and HAM damage

**Fig. 2:**
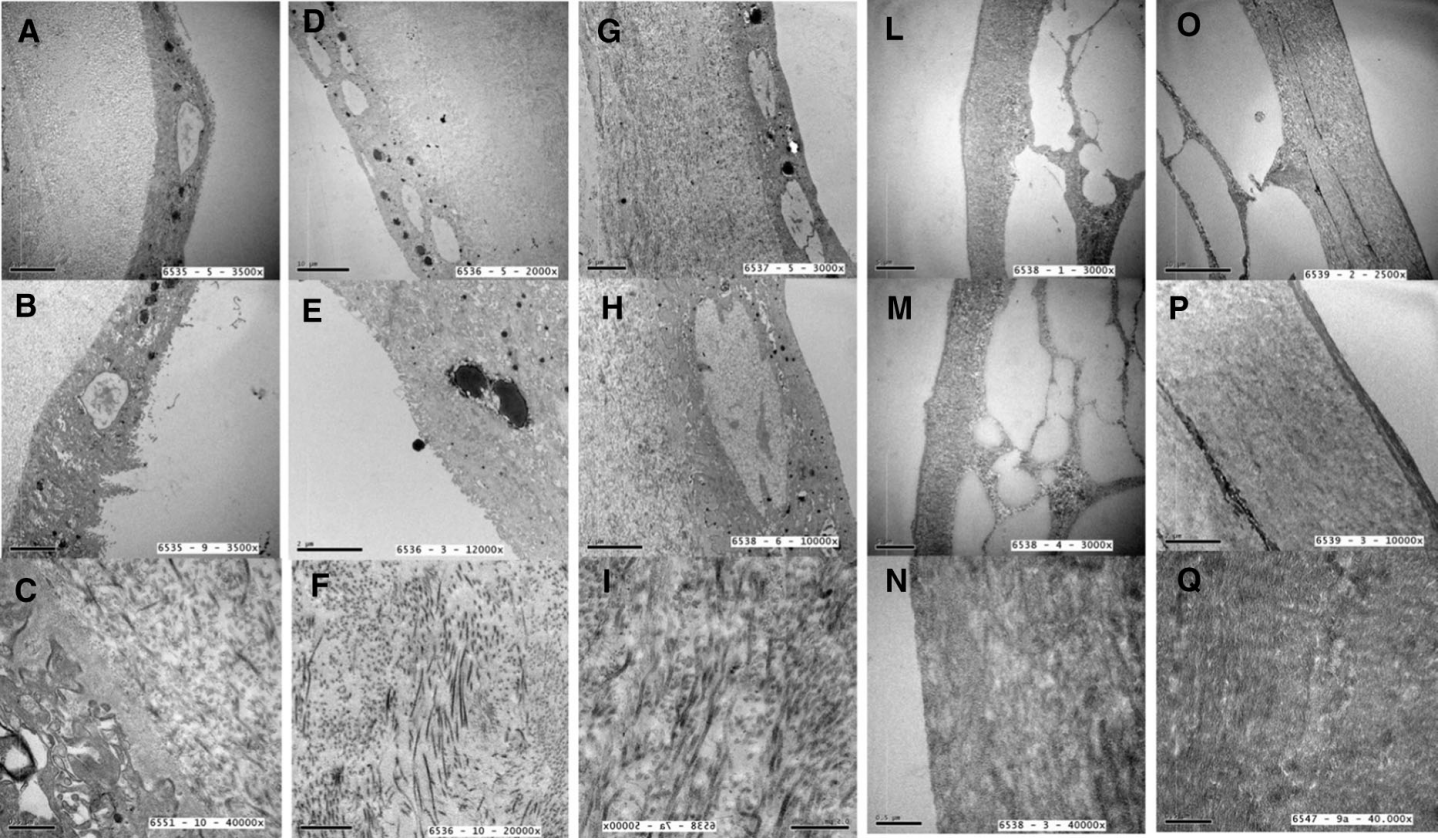
Ultrastructure evaluation of differently prepared HAM samples: **a**, **b**, **c** fresh-frozen HAM, **d**, **e**, **f** freeze-dried HAM, **g**, **h**, **i** freeze-dried HAM, 10 kGy irradiated, **l**, **m**, **n** freeze-dried HAM, 20 kGy irradiated, **o**, **p**, **q** freeze-dried HAM, 30 kGy irradiated

Figure 2 shows representative ultrastructural images of different HAM samples. The transmission electron microscopy images in Fig. 2a–c show fresh-frozen HAM samples to have well-preserved epithelium, with the presence of apical microvilli, cytoplasmic vacuoles and basement membrane. Electrondense structures and hemidesmosomes are also visible. The collagen matrix morphology of the basal lamina is also fairly well preserved. In the images Fig. 2d–f, taken after freeze-drying, the epithelium, microvilli, vacuoles, electron-dense structures, basement membrane, and hemidesmosomes are still visible. Nuclear changes can be seen while the collagen matrix morphology of the basal lamina is largely preserved. One sample (Fig. 2d) shows more severe tissue damage, with the epithelium and basement membrane no longer visible. Samples exposed to 10 kGy irradiation (Fig. 2g–i) display surface epithelium with loss of microvilli, intracytoplasmic vacuoles, electron-dense structures and nuclear degenerative changes. The basement membrane also appears thinner and there are fewer hemidesmosomes. However, the collagen matrix of the lamina densa is preserved. In one sample (Fig. 2g), the changes are more severe: no epithelium or basement membrane is visible and the collagen matrix of the lamina densa is degenerated and markedly disrupted. The three images of samples exposed to 20 kGy irradiation (Fig. 2l–n) evidence no epithelium or basement membrane in two of the three specimens. The collagen matrix of the lamina densa is poorly preserved and almost degenerated. In the image of the third 20 kGy-irradiated sample (Fig. 2m), the epithelium, cytoplasmic vacuoles and electron-dense structures are partially preserved, but degenerative nuclear changes can be observed. However, the basement membrane and hemidesmosomes are preserved. 2 of the 3 samples exposed to 30 kGy irradiation (Fig. 2o–q) show no epithelium or basement membrane, whereas one sample evidences only a thinning of these structures; the collagen matrix of the lamina densa is, however, thinner and abnormal. Almost complete homogenization of the cell surface layer can be seen in two examples.

## Discussion

Our findings show that all the cytokines analyzed were present in fresh-frozen samples and were still present after freeze-drying, whereas sterilization of HAM by exposure to γ-radiation led to significant cytokine losses. Moreover, sterilization by γ-irradiation proportionally affected the ECM ultrastructure, indicative that the higher the irradiation dose, the more severe the ECM damage.

The cytokines analyzed in this study are among those most frequently indicated in the literature as involved in wound healing and tissue regeneration processes.

TIMP-1, -2 and -4 are inhibitors of matrix metalloproteinases (MMPs), a group of peptidases involved in degradation of the ECM. In addition to their inhibitory role, TIMPs promote cell proliferation in a wide range of cell types, and may also have an anti-apoptotic function. Basic FGF is a potent angiogenic factor and an endothelial cell mitogen, and has been described as a multipotent cytokine regulating cell growth and differentiation, matrix composition, chemotaxis, cell adhesion and migration in a variety of cell types (Makino et al. 2010). bFGF is known to stimulate proliferation of cultured fibroblasts.

Members of the PDGF family are mitogenic factors for cells of mesenchymal origin. PDGF-BB modulates endothelial proliferation and angiogenesis (Battegay et al. 1994), while IL-10, secreted by macrophages and mast cells, is an important immunoregulatory cytokine with anti-inflammatory effects. IL-10 is also released by cytotoxic T cells to inhibit viral infection (Khan 2008). TGF-β1 and EGF both play an important role in the growth, proliferation and differentiation of numerous cell types. In particular, EGF is a potent mitogen for epithelial cell growth, promoting wound healing following transplantation (Koizumi et al. 2000).

Our results showed the various TIMPs to have differing sensitivity to gamma-irradiation. This may be due to several reasons, including, for example, being part of a protein complex, which reduces the likelihood of being affected by radiation. The different amino acid content of the three proteins may also contribute to determining sensitivity to radiation. In this regard, one of the main targets of radiation is the amino acid tyrosine. Interestingly, TIMP-4 contains twice as much tyrosine as TIMP-1 and -2, which may explain why TIPM-4 is more sensitive to radiation treatment than TIMP-1/-2. It is also possible, however, that gamma irradiation directly affects the immunogenic structure of TIMP-4 and EGF, disrupting specific epitope(s) recognized by the antibodies used in the ELISA Kit. A similar effect may also be present in other cytokines. However, if the affected epitope(s) is not recognized by the kit antibodies, no difference in protein content will detected. It should also be noted that the experiments conducted were confined to detecting the presence of the proteins and not their biological function. Differences in the observed concentration of the various TIMPs might not reflect actual biological activity. The same hypothesis also applies to the other cytokines, such as EGF, whose levels fell significantly following radiation treatment.

While the literature reports many studies describing HAM composition after different preservation procedures, the many differences in HAM harvesting and processing methods make comparisons almost impossible. Hao et al. demonstrated that HAM epithelial and mesenchymal cells cryopreserved in glycerol at −80 °C express interleukin-1 receptor antagonist, all four TIMPs, collagen XVIII, and interleukin-10 (Hao 2000). Other authors used the same preservation method and found HAM to contain EGF, TGFα, KGH, HGF, bFGF, TGF-β1, and -β2 (Li et al. 2005). Another paper analyzed EGF, HGF, FGF, and TGF-β1 content in a tissue-suspension obtained from frozen, freeze-dried, powdered and γ-irradiated HAM, reporting that the freeze-drying process causes a reduction in total protein compared to freezing alone, while powdering causes a significantly increased release of EGF (Russo et al. 2012). Lim et al. compared decellularized and dehydrated human amniotic membrane with cryopreserved human amniotic membrane, and reported significant differences in the composition and ultrastructure of dehydrated HAM as shown by histological and immunohistochemical examination (Lim et al. 2010). Nakamura et al. reported no statistically significant differences in the physical strength of cryopreserved HAM or freeze-dried HAM treated with γ-irradiation. The authors also observed no significant alterations in tissue structure or ECM components (Nakamura et al. 2004).

Ultrastructural analysis provided additional evidence of the damage caused by γ-rays, in contrast to the absence of any severe damage evidenced in fresh-frozen and freeze-dried samples. γ-irradiation induced major damage to the epithelium, basement membrane and lamina densa, which was more severe after exposure to 20 and 30 kGy γ-irradiation.

Preservation of the epithelium structure is of major importance since epithelial cells express key anti-inflammatory factors as reported by Hao (2000).

Exposure to γ-radiation is known to induce cellular and sub-cellular damage. Radiation has a direct effect, interacting with the structures of the target to cause ionization and subsequent biological changes (Valentin 2005; Lehnert 2007) and also an indirect action, that can lead to the production of Reactive Oxygen Species (ROS), which in turn, may induce important membrane changes and cellular injury, with an increase in polarization at higher radiation doses (>3 kGy; Mishra 2004).

In 2014, Hamid et al. demonstrated changes to the cell morphology of glycerol-preserved amnion exposed to 35 kGy, while air-dried HAM underwent changes at 25 kGy. and concluded, that cell structure preservation of glycerol-preserved amnion after radiation is probably due to the radio-protectant properties of glycerol, which removes water and limits the formation of free radicals (Ab Hamid et al. 2014).

In our study, we observed that γ-radiation causes important changes in the amniotic epithelium, basal lamina and basement membrane. In addition, we detected the loss of important cytokines necessary to promote wound healing and epithelialization, inhibit fibrosis and scarring, and regulate angiogenesis. In contrast, we also demonstrated that cytokine levels and the amniotic structure-key features responsible for the favorable clinical outcomes obtained with HAM-were well preserved only in fresh-frozen and freeze-dried HAM samples.

In conclusion, processing the amniotic membrane under sterile conditions to guarantee safety at every step as an alternative to final sterilization with γ-irradiation is strongly recommended in order to avoid alteration of the biological characteristics of the amniotic membrane.
